# Impairment of proteasome-associated deubiquitinating enzyme Uchl5/UBH-4 affects autophagy

**DOI:** 10.1242/bio.061644

**Published:** 2025-02-06

**Authors:** Sweta Jha, Johanna Pispa, Carina I. Holmberg

**Affiliations:** Department of Biochemistry and Developmental Biology, Medicum, Faculty of Medicine, University of Helsinki, 00290, Helsinki, Finland

**Keywords:** Autophagy, Autophagosome, Autolysosome, Deubiquitinating enzyme (DUB), Proteasome-associated DUBs, Ubiquitin-proteasome system, Tissue specificity

## Abstract

The autophagy–lysosomal pathway (ALP) and the ubiquitin–proteasome system (UPS) are the two major intracellular proteolytic systems that mediate protein turnover in eukaryotes. Although a crosstalk exists between these two systems, it is still unclear how UPS and ALP interact *in vivo*. Here, we investigated how impaired function of the proteasome-associated deubiquitinating enzyme (DUB) Uchl5/UBH-4 affects autophagy in human cells and in a multicellular organism. We show that downregulation of *Uchl5* by siRNA reduces autophagy by partially blocking the fusion of autophagosomes with the lysosomes in HeLa cells, which is similar to a previously reported role of the proteasome-associated DUB Usp14 on autophagy. However, exposure of *Caenorhabditis elegans* to *ubh-4* or *usp-14* RNAi, or to their pharmacological inhibitors*,* results in diverse effects on numbers of autophagosomes and autolysosomes, without blocking the lysosomal fusion, in the intestine, hypodermal seam cells and the pharynx. Our results reveal that impairment of Uchl5/UBH-4 and Usp14 affects autophagy in a tissue context manner. A deeper insight into the interplay between UPS and ALP in various tissues *in vivo* has the potential to promote development of therapeutic approaches for disorders associated with proteostasis dysfunction.

## Teaser

**Summary:** Modulation of UPS via pharmacological or genetic impairment of the proteasome-associated DUB Uchl5/UBH-4 affects autophagy in human cells and in a tissue-differential manner in *C. elegans*.

## INTRODUCTION

Protein homeostasis is a dynamic balance between the production and degradation of proteins and is essential for cell survival and growth. In eukaryotes, the turnover of proteins is facilitated by two main intracellular proteolytic systems: the autophagy–lysosomal pathway (ALP) (hereafter referred to as autophagy), and the ubiquitin–proteasome system (UPS). These two proteolytic systems are essential components of the cellular protein quality control system, but also crucial for the maintenance of the amino acid pools and energy balance (reviewed in Schreiber and Peter, 2014 and Pohl and Dikic, 2019).

Autophagy is a highly regulated proteolytic pathway that is well conserved from yeast to humans. It is responsible for degradation of mainly long-lived proteins, cytoplasmic organelles, and other cellular components by delivering them to the lysosomes, and the breakdown products are reused for cellular processes (reviewed in Wen and Klionsky, 2020). The autophagy process starts with the formation of an isolation membrane, the phagophore, which elongates to engulf the substrate(s) and forms the double-layered autophagosome. Autophagosomes then fuse with late endosomes and lysosomes, which leads to the formation of autolysosomes, where the substrate(s) is(are) degraded by lysosomal hydrolases (Kametaka et al., 1998; Mizushima, 2007, 2018; Sun-Wang et al., 2020). Autophagy is mediated through the conserved action of the Atg protein family, of which Atg8, a ubiquitin-like protein, is a key participant in the autophagic process in yeast. Atg8 and its mammalian homologs GABARAP and LC3 play a crucial role in various stages of autophagy, encompassing initiation, cargo recognition and engulfment, as well as the closure of autophagosome (Mizushima et al., 1998; Nakatogawa et al., 2007; Knorr et al., 2014; Klionsky et al., 2021). At the autophagosome membrane, Atg8/GABARAP/LC3 is conjugated with phosphatidylethanolamine (PE), and the Atg8-PE/LC3-II serves as a well-established marker for assessment of autophagy (Nakatogawa et al., 2007; reviewed in Klionsky et al., 2021 and Yamamoto et al., 2023). In a multicellular organism, a role of autophagy was firstly reported in *Caenorhabditis elegans* dauer development (Meléndez et al., 2003) and autophagy has since been shown to be essential also for embryogenesis, longevity, and stress responses (Zhao et al., 2009; Tian et al., 2010; Palmisano and Meléndez, 2019; Meléndez et al., 2003; Alberti et al., 2010; Wu et al., 2015; Chang et al., 2017). *C. elegans* has two Atg8 homologs, i.e., LGG-1 and LGG-2, corresponding to GABARAP and LC3, respectively. Both LGG-1 and LGG-2 localize to autophagosomes and LGG-1 is required for the recruitment of LGG-2 (Alberti et al., 2010; Manil-Ségalen et al., 2014). The structural conservation of LGG-1/GABARAP and LGG-2/LC3 emphasizes their pivotal role in autophagy during developmental stages, longevity, and responses to stress (Meléndez et al., 2003; Alberti et al., 2010; Wu et al., 2015; Chang et al., 2017). Recently, a lipidation-independent role of LGG-1 in autophagy was reported (Leboutet et al., 2023).

UPS is the primary proteolytic pathway responsible for degradation of soluble, short-lived, and misfolded proteins in the cytoplasm and nucleus. UPS-mediated proteolytic degradation is a multistep process, where polyubiquitination of proteasomal substrates occurs through the action of a cascade of three different classes of enzymes: ubiquitin-activating enzymes (E1), ubiquitin-conjugating enzymes (E2) and ubiquitin ligases (E3). The polyubiquitinated substrates are then degraded by the evolutionarily conserved 26S proteasome, a large ATP-dependent multicatalytic protease complex (Chen et al., 2020; reviewed in Finley, 2009; Dikic, 2017; Raffeiner et al., 2023). Prior to degradation, ubiquitin chains are removed from the substrates by three proteasome-associated deubiquitinating enzymes (DUBs); the cysteine proteases UchL5/Uch37 (Stone et al., 2004) and Usp14 (Borodovsky et al., 2001), and the metalloprotease Rpn11 (Verma et al., 2002). Of these proteasome-associated DUBs, Rpn-11 is a subunit of the proteasome, whereas Uchl5 and Usp14 bind to the proteasome (reviewed in Collins and Goldberg, 2017; Finley, 2009; and Kocaturk and Gozuacik, 2018). Rpn11 is responsible for *en bloc* removal of ubiquitin chains. Usp14 functions either by trimming or by *en bloc* removal of ubiquitin chains, and Uchl5 functions by trimming or by debranching of polyubiquitin chains (Hanna et al., 2006; Finley, 2009; Lee et al., 2011, 2016; Deol et al., 2020). Due to their modes of action Usp14 and Uchl5 affect the kinetics and affinity of the substrate-proteasome interaction, thereby influencing the proteolytic capacity of the proteasome. Impaired Usp14 or Uchl5 function has been reported to enhance proteasomal degradation of substrates (Lam et al., 1997; Hanna et al., 2006; Koulich et al., 2008; Matilainen et al., 2013; Kim and Goldberg, 2017, 2018; Lee et al., 2018; Deol et al., 2020), and also to cause accumulation of proteasomal substrates (Lee et al., 2018; Chadchankar et al., 2019).

In the past, autophagy and UPS were believed to function as independent systems targeting distinct substrates, however, accumulating evidence describes interactions between these two systems including some shared substrates (Pohl and Dikic, 2019; Raffeiner et al., 2023). This interplay is not always a direct compensatory mechanism and exhibits complexity and diversity. Studies on pharmacological and genetic impairment of autophagy have reported contrasting outcomes, as both inhibition of proteasomal degradation as well as upregulation of proteasome activity have been reported. Ding et al. have shown that siRNA knockdown of Atg6/Beclin1 or Atg8/LC3 results in accumulation of polyubiquitinated proteins in HCT116 human colorectal carcinoma cells (Ding et al., 2007). Additionally, impairment of autophagy by siRNA or chemical treatments in HeLa cervical cancer cells or by Atg5 knockout in mouse embryonic fibroblasts (MEF) cells induces accumulation of a UPS reporter due to decreased proteasomal degradation (Korolchuk et al., 2009). In neuroblastoma cells, inhibition of lysosomal function has been reported to decrease proteasome activity (Qiao and Zhang, 2009). However, there are also studies showing that autophagy modulation has an opposite effect on UPS. Wang et al. have demonstrated that inhibition of autophagy via chemical treatments or downregulation of Atg5 or Atg7 leads to upregulation of proteasome activity and increased expression of proteasomal subunits in colon cancer cell lines SW1116 and HCT116 (Wang et al., 2013). Kim et al. have shown that autophagy-defective Atg5 knockout MEF cells increased proteasome activity and that starvation-induced autophagy resulted in decreased proteasome activity without affecting the stability of proteasome subunits in HEK293 human embryonic kidney cells (Kim et al., 2018). We have previously shown that downregulation of various autophagy genes by RNAi does not result in systemic upregulation of UPS function in *C. elegans* but depending on the target gene the outcome on UPS function or proteasome expression varies in a tissue-specific manner (Jha and Holmberg, 2020). Several studies on impaired proteasomes have reported an induction of autophagy in human cell lines, mice cells and in *Drosophila* (Zheng et al., 2011; Shen et al., 2013; Lőw et al., 2013; Fan et al., 2016; Li et al., 2019).

We have previously shown that Uchl5 depletion enhances proteasomal substrate degradation in human U-2 OS (osteosarcoma) cells and that the *C. elegans* homolog UBH-4 regulates proteasome activity in *C. elegans* intestine, and affects the lifespan and health span of the animal (Matilainen et al., 2013). Here, we investigated the effect of Uchl5/ UBH-4 on autophagy in human cells and *C. elegans*. We also performed comparison studies between Uchl5 and Usp14, as *Usp14* downregulation has been shown to decrease autophagic flux in HEK293 and MEF cells (Kim et al., 2018). Our data reveal that *Uchl5 and Usp14* siRNA treatments or pharmacological inhibition reduce autophagy by blocking autophagosome–lysosome fusion in GFP-LC3-RFP-LC3ΔG HeLa cells. When investigating the impact of these DUBs on autophagy at the tissue level, our results show that downregulation of *ubh-4* and *usp-14* by RNAi or by using pharmacological inhibitors causes differential autophagic responses in the intestine, hypodermal seam cells and the pharynx in *C. elegans*. Our studies highlight the complexity in the interaction between the UPS and autophagy in a multicellular organism.

## RESULTS

### Knockdown of deubiquitinating enzyme (DUB) *Uchl5* reduces autophagic flux

To investigate how the proteasome–associated deubiquitinating enzyme (DUB) *Uchl5* affects autophagy, we downregulated *Uchl5* by siRNA and analyzed the effect on autophagic flux. We used the previously developed HeLa cell line expressing the autophagy reporter GFP-LC3-RFP-LC3ΔG, which is cleaved into equimolar amounts of GFP-LC3 and RFP-LC3ΔG by the endogenous protease ATG4 in the cell (Kaizuka et al., 2016). While phosphatidylethanolamine-conjugated GFP-LC3 (LC3-II) in the autophagosomes is degraded upon fusion with lysosomes, the stable RFP-LC3ΔG present in the cytoplasm serves as an internal control, and thus, the fluorescence ratio of the GFP/RFP signal reversely correspond to autophagic activity (Kaizuka et al., 2016) (Fig. S1A). For functional validation of the reporter cell lines, we treated the cells with Bafilomycin A (BAFA), which inhibits autophagosome-lysosome fusion blocking LC3-II turnover by acidic hydrolases in autolysosomes, and observed the expected increase in GFP/RFP ratio (Fig. S2A) and accumulation of LC3-II and p62 (Fig. S2B), as previously reported (Redmann et al., 2017; Kaizuka et al., 2016).

**Fig. 1.**
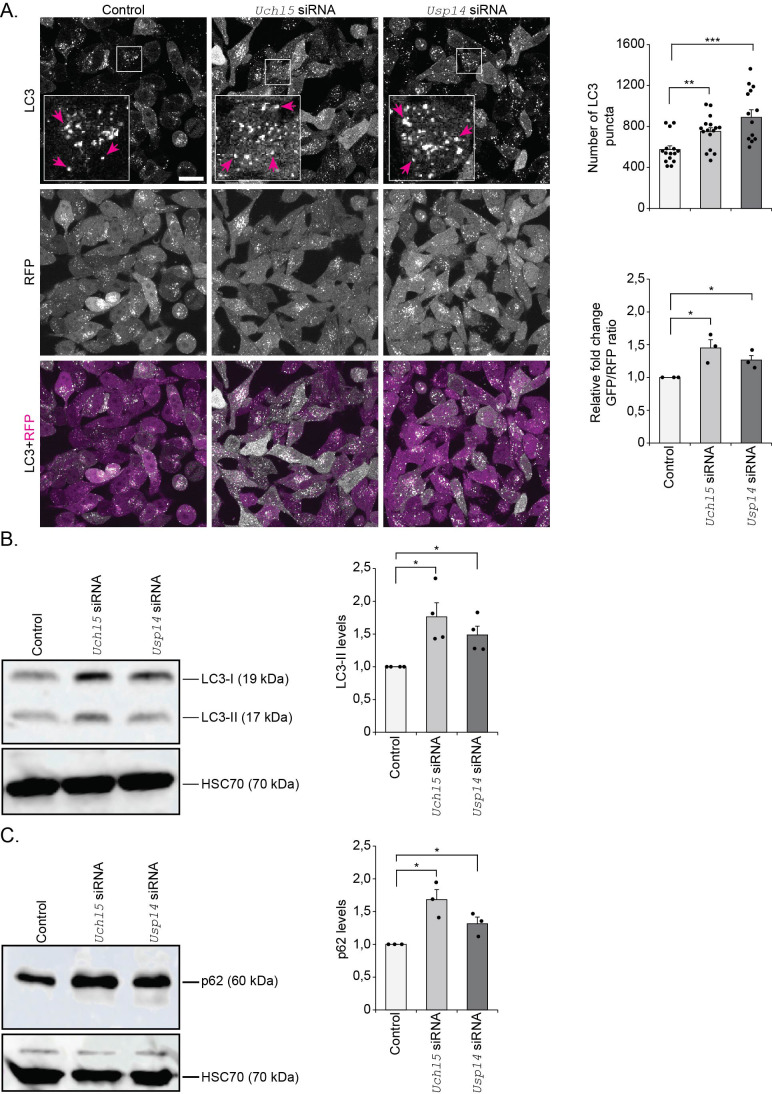
**Downregulation of proteasome-associated DUBs *Uchl5* and *Usp14* reduces autophagy.** (A) Fluorescence confocal images of control, *Uchl5* or *Usp14* siRNA-treated GFP-LC3-RFP-LC3ΔG HeLa cells 48 h post-transfection. Insets show enlarged view of the indicated areas. Magenta arrows point to some of the GFP-LC3 puncta. Scale bar: 20 µm. The right upper graph shows the quantification of the number of GFP-LC3 puncta per image. The right lower graph shows quantification of the relative fold change in the ratio of GFP to RFP per image (control set at 1). Results are from three independent experiments (a total of 15-17 images per treatment were analyzed). Error bars, s.e.m., *P<0.05, **P<0.01, ***P<0.001 compared to control. (B,C) GFP-LC3-RFP-LC3ΔG HeLa cells treated with control, *Uchl5* or *Usp14* siRNA for 48 h. Whole cell extracts were analyzed by SDS-PAGE and immunoblotted against LC3-II, p62 and HSC70. The graphs on the right show average fold change in levels of LC3-II (B) and p62 (C) normalized against HSC70. Results are the mean of quantifications from 3-4 independent experiments. Error bars, s.e.m., *P<0.05 compared to the control (set as 1). Statistical analyses were performed from raw data.

**Fig. 2.**
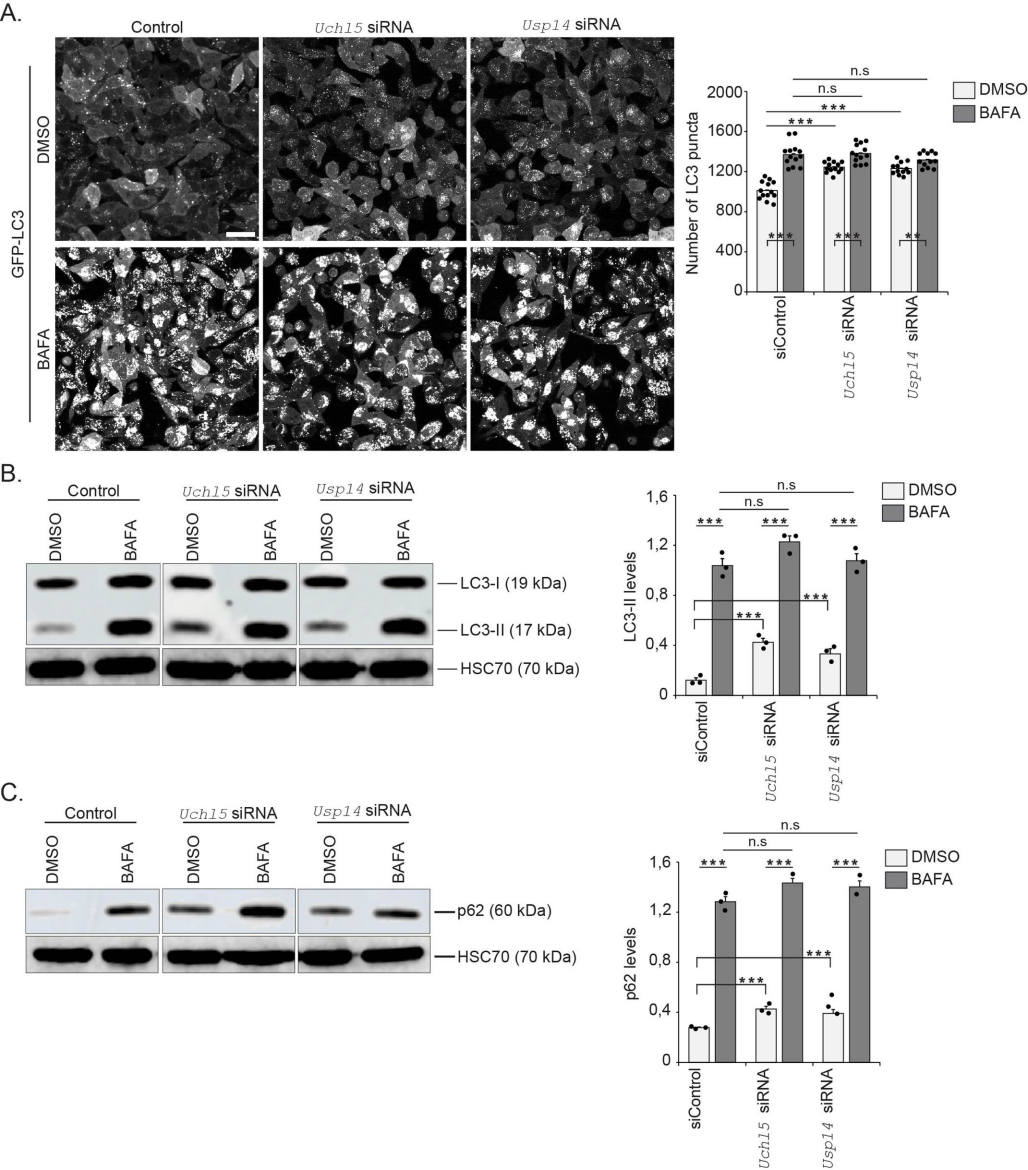
**Downregulation of *Uchl5* or *Usp14* reduces autophagy due to blockage of autophagosome–lysosome fusion.** (A) Fluorescence confocal images of control, *Uchl5* or *Usp14* siRNA-treated GFP-LC3-RFP-LC3ΔG HeLa cells 48 h post-transfection and treated with DMSO or BAFA (100 nM) for the last 6 h. Scale bar: 20 µm. The right graph shows the quantification of the number of GFP-LC3 puncta per image. Results are from three independent experiments (a total of 15-18 images per treatment were analyzed). Error bars, s.e.m., **P<0.01, ***P<0.001 compared to respective control or a treatment. (B,C) GFP-LC3-RFP-LC3ΔG HeLa cells treated with control, *Uchl5* or *Usp14* siRNA for 48 h and treated with DMSO or BAFA for the last 6 h. Whole-cell extracts were analyzed by SDS-PAGE and immunoblotted against LC3-II, p62 and HSC70. The samples were from the same experiment, but run on the gel in a different order. The graphs (on right panels) show average fold change in levels of LC3-II (B) and p62 (C) normalized against HSC70. Results are the mean of quantifications from three independent experiments. Error bars, s.e.m., ***P<0.001, n.s (not significant) compared to the respective control or a treatment. Statistical analyses were performed from raw data.

Treatment of GFP-LC3-RFP-LC3ΔG HeLa cells with *Uchl5* siRNA resulted in reduced *Uchl5* mRNA and Uchl5 protein levels, respectively (Fig. S1B,C). We also downregulated the proteasome-associated DUB *Usp14* (Fig. S1B,C), the inhibition of which has previously been shown to impair autophagy at the autophagosome-lysosome fusion step in HEK293 and MEF cells (Kim et al., 2018), to enable comparison of autophagy studies with Uchl5. Both *Uchl5* and *Usp14* siRNA treatments resulted in an increased number of GFP-LC3 puncta in the HeLa cells, as measured by live imaging, and an increase in the GFP/RFP ratio was detected, indicating inhibition of autophagic activity (Fig. 1A). Next, we checked the levels of LC3 in cell lysates upon *Uchl5* or *Usp14* knockdown and observed a significant accumulation of LC3-II in these cells (Fig. 1B). Similarly, we detected an accumulation of the autophagy marker/substrate p62 upon *Uchl5* or *Usp14* siRNA treatment (Fig. 1C). To investigate whether *Uchl5* or *Usp14* knockdown causes a block in autophagy or potentially affect the number of lysosomes, we first performed immunofluorescence analysis against lysosome-associated membrane protein 2 (LAMP2). We detected no change in LAMP2 fluorescence intensity showing that the number of lysosomes was not affected upon *Uchl5* or *Usp14* downregulation (Fig. S3). We next checked the effect of *Uchl5* or *Usp14* knockdown on autophagic flux by exposing the cells to BAFA treatment and performing live imaging of GFP-LC3. BAFA treatment increased the number of GFP-LC3 puncta in control cells as well as in *Uchl5* or *Usp14* siRNA-treated cells (Fig. 2A). However, no further enhancement in the BAFA-induced GFP-LC3 puncta formation was observed in the *Uchl5* or *Usp14* siRNA-treated cells compared to the control cells (Fig. 2A), revealing that downregulation of *Uchl5* or *Usp14* cause a clear reduction, but not a complete block of autophagosome–lysosome fusion. Similar to the live image results, Western blot analysis of LC3-II and p62 upon BAFA treatment showed no difference between control and *Uchl5* or *Usp14* siRNA-treated cells (Fig. 2B,C). Taken together, our live cells and *in vitro* results reveal that *Uchl5* and *Usp14* downregulation decreases autophagy by blocking fusion of autophagosomes with lysosomes in HeLa cells.

### Pharmacological inhibition of proteasome-associated DUBs decreases autophagy

**Fig. 3.**
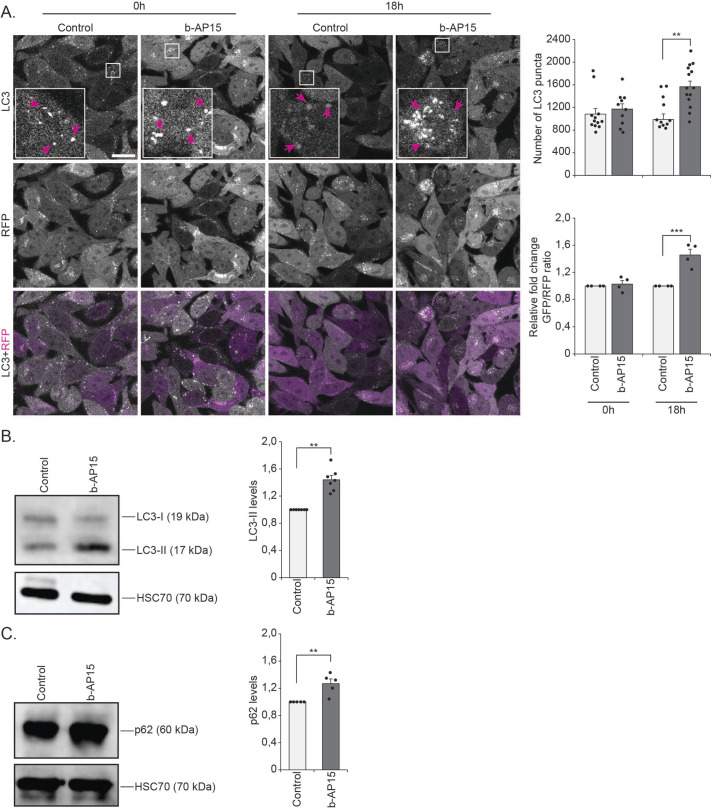
**Pharmacological inhibition of proteasome-associated DUBs Uchl5 and Usp14 reduces autophagy.** (A) Fluorescence confocal images of control (DMSO) or b-AP15 (1 µM) treated GFP-LC3-RFP-LC3ΔG HeLa cells after 18 h of treatment. Insets show enlarged view of the indicated areas. Magenta arrows point to some of the puncta. Scale bar: 20 µm. The right upper graph shows the quantification of the number of GFP-LC3 puncta per image. The right lower graph shows the quantification of the relative fold change in the ratio of GFP to RFP per image (control set at 1). Results are from four independent experiments (a total of 15-20 images per treatment were analyzed). Error bars, s.e.m., **P<0.01, ***P<0.001 compared to control. (B,C) GFP-LC3-RFP-LC3ΔG HeLa cells treated with control (DMSO) or b-AP15 (1 µM) for 18 h. Whole-cell extracts were analyzed by SDS-PAGE and immunoblotted against LC3-II, p62 and HSC70. The graphs (on right panel) show average fold change in levels of LC3-II (B) and p62 (C) normalized against HSC70. Results are the mean of quantifications from 5-7 independent experiments. Error bars, s.e.m., **P<0.01 compared to the control (set as 1). Statistical analyses were performed from raw data.

To complement the studies on genetic downregulation of *Uchl5* and *Usp14*, we also investigated the effect of pharmacological inhibition of these two proteasome-associated DUBs in the GFP-LC3-RFP-LC3ΔG HeLa cells. Of the commonly used DUB inhibitors, b-AP15 blocks the DUB activity of both Uchl5 and Usp14 (D'Arcy et al., 2011) and IU1 inhibits specifically Usp14 activity (Lee et al., 2010). To our knowledge, there is so far no specific inhibitor for Uchl5 and, therefore, we performed comparison analysis between dual inhibition of Uchl5 and Usp14 and single inhibition of Usp14 to reveal insight on the action of Uchl5 on autophagy. We investigated autophagy in GFP-LC3-RFP-LC3ΔG HeLa cells treated with b-AP15 or IU1, and the number of GFP-LC3 puncta as well as the GFP/RFP ratio were analyzed. We detected that b-AP15 treatment increased the number of GFP-LC3 puncta as well as the GFP/RFP ratio (Fig. 3A). The b-AP15 treatment also resulted in increased amount of LC3-II (Fig. 3B) and p62 (Fig. 3C), as analyzed by Western blotting. These results are indicative of reduced autophagy and correlate with the reduced autophagy we detected upon *Uchl5* or *Usp14* downregulation by siRNA (Fig. 1). Similarly, treatment with IU1 resulted in increased number of GFP-LC3 puncta and of GFP/RFP ratio (Fig. S4A), as well as an accumulation of both LC3-II and p62 (Fig. S4B,C), indicating a reduction of autophagy in GFP-LC3-RFP-LC3ΔG HeLa cells, which is consistent with a previous report (Kim et al., 2018). Taken together, our results reveal that both downregulation and pharmacological inhibition of Uchl5, as well as Usp14, cause reduction of autophagy.

### Differential autophagic tissue responses to downregulation of the proteasome-associated DUBs *ubh-4* and *usp-14* in a multicellular organism *C. elegans*

**Fig. 4.**
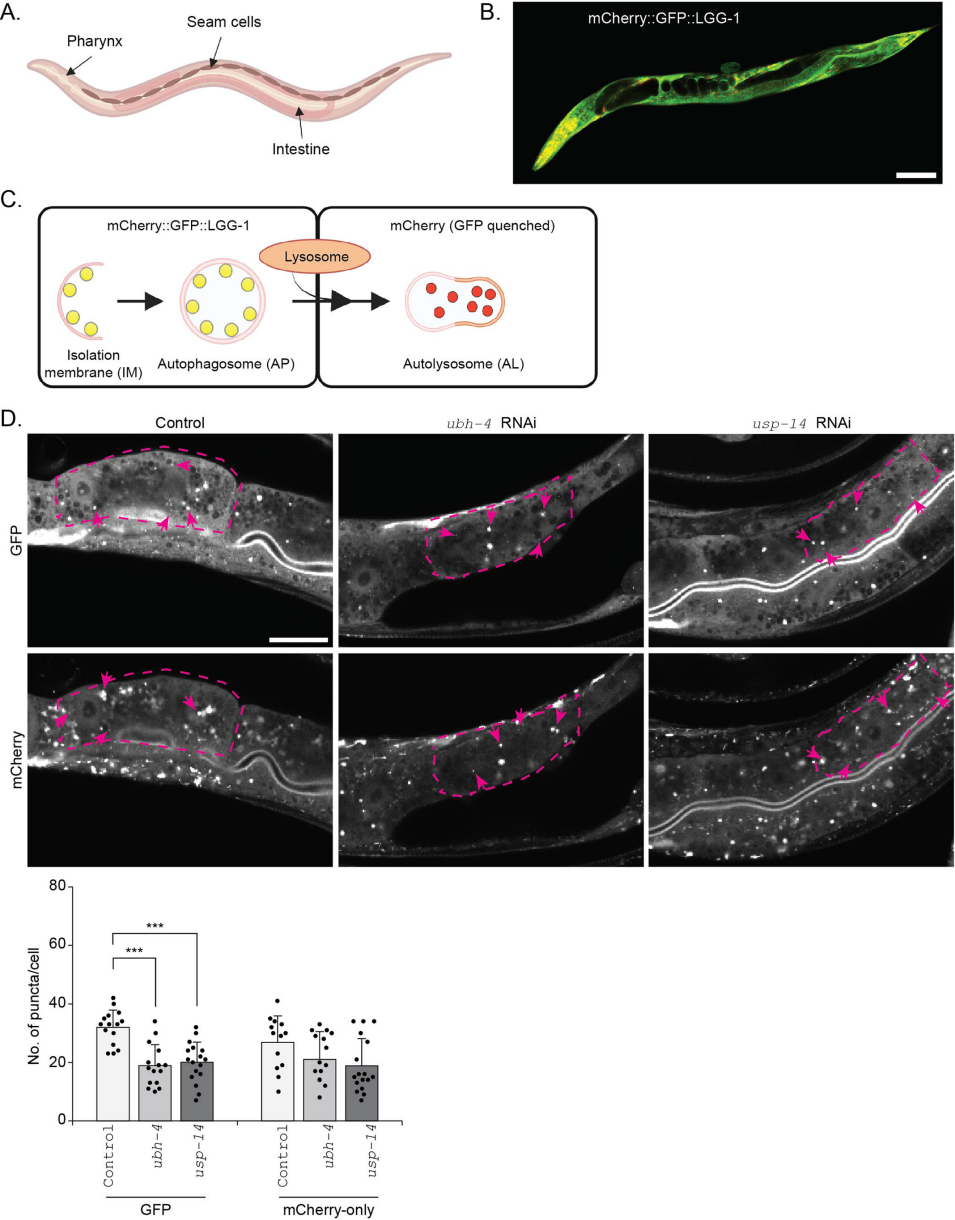
**The autophagosome pool size decreases upon downregulation of the proteasome-associated DUBs *ubh-4* and *usp-14* in intestinal cells of *C. elegans***. (A) Schematic representation of the tissues (studied in this article) of an adult *C. elegans.* (B) Fluorescence image of a 1-day-old animal expressing mCherry::GFP::LGG-1. Scale bar: 100 µm. Note that the image was taken with different setting for GFP and mCherry. (C) Schematic representation of the fluorescence states of mCherry::GFP::LGG-1 at the different stages of autophagy (Isolation membrane, IM; Autophagosome, AP; Autolysosome, AL). (D) Representative confocal micrographs of control, *ubh-4* or *usp-14* RNAi-treated mCherry::GFP::LGG-1 animals at day 1 of adulthood. Individual intestinal cells are outlined with magenta dashed lines and the magenta arrows point to some of the puncta. Scale bar: 50 µm. Note that the mCherry was imaged with lower gain setting. The graph (below) shows the quantification of the number of puncta positive for GFP and mCherry-only in individual intestinal cells. Results are from three independent experiments. Puncta were counted from a total of 15-18 individual intestinal cells from 12-15 animals (all distinct puncta of variable sizes were counted). Error bars, STD, ***P<0.001 compared to control.

Previously, we have reported that impairment of autophagy affects proteasome function differently in the intestine and body-wall muscle in *C. elegans* (Jha and Holmberg, 2020). To address whether downregulation of proteasome-associated DUBs *ubh-4*, the *uchl5* homolog, and *usp-14* induces a tissue-specific or systemic effect on autophagy in *C. elegans*, we downregulated *ubh-4* and *usp-14* by RNAi-feeding and analyzed autophagy in intestinal cells, hypodermal seam cells and pharynx (Fig. 4A). These cell types were chosen as they have previously been used for assessing autophagy in *C. elegans* (Zhang et al., 2015; Chang et al., 2017). The RNAi treatment resulted in efficient reduction of *ubh-4* and *usp-14* mRNA levels, as measured in whole animal lysates (Fig. S5). For monitoring autophagy, we used the previously established dual-fluorescent marker strain expressing mCherry::GFP::LGG-1 driven by the endogenous *lgg-1* promoter (Chang et al., 2017) (Fig. 4B). In these animals, the autophagosomes (APs) are indicated by puncta positive for both GFP and mCherry, and the autolysosomes (ALs) are visualized as red puncta (mCherry) due to lysosomal quenching of GFP (Chang et al., 2017) (Fig. 4C).

**Fig. 5.**
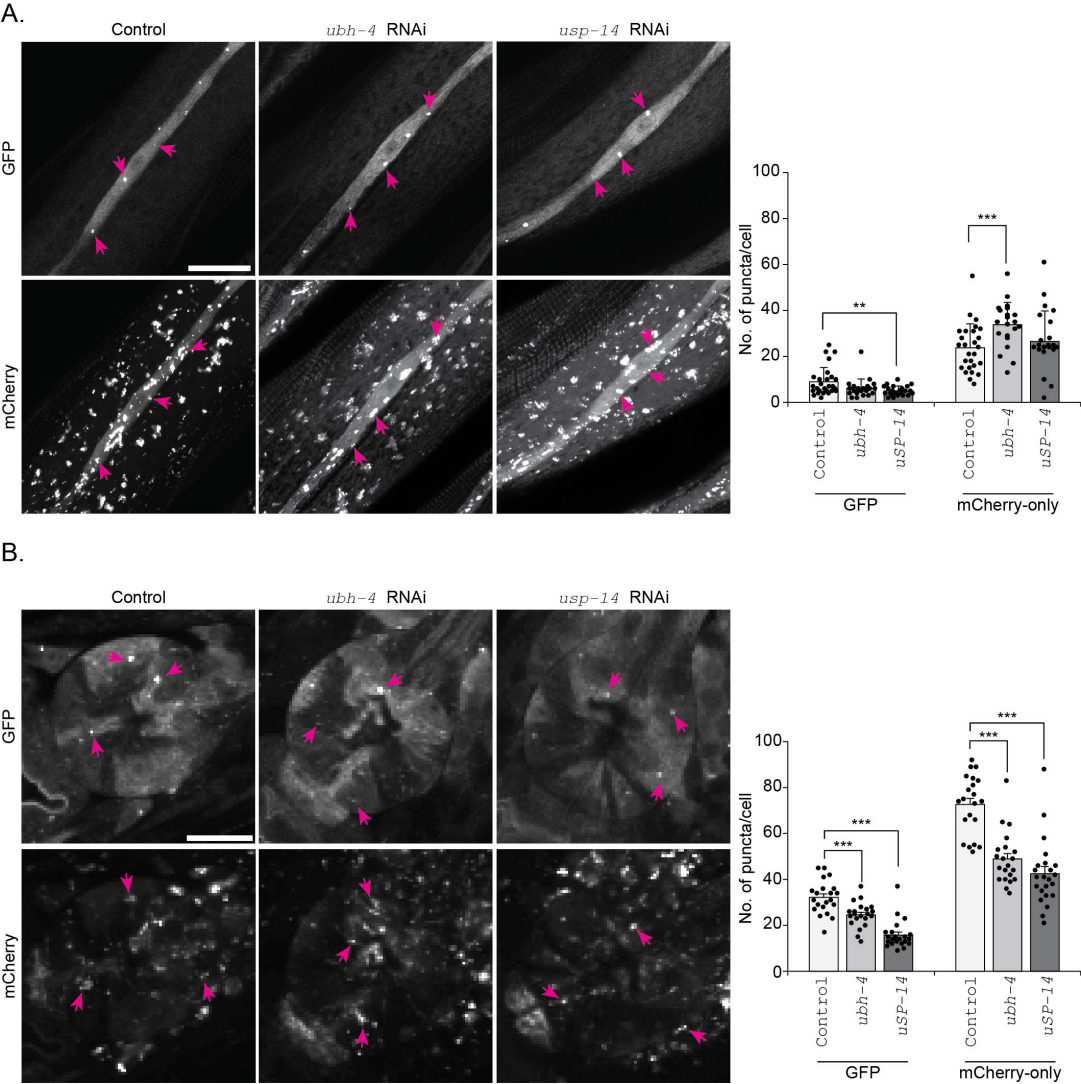
**Downregulation of the proteasome-associated DUBs *ubh-4* and *usp-14* affects autophagy differently in the pharynx and in hypodermal seam cells.** Fluorescence confocal micrographs of control, *ubh-4* or *usp14* RNAi-treated mCherry::GFP::LGG-1 animals showing hypodermal seam cells (A) and, the pharynx (B). Graphs show the quantification of the number of puncta positive for GFP and mCherry-only. Results are from five independent (for hypodermal seam cells) or three independent [for pharynx (second generation)] experiments. Puncta were counted from a total of 20-25 pharynges from 20-25 animals and 25-30 hypodermal seam cells from 20-25 animals. Error bars, STD, **P<0.01 and ***P<0.001 compared to control.

We started the RNAi feeding at L1 larval stage, imaged live animals at day 1 of adulthood, and subsequently manually counted fluorescent puncta in individual intestinal cells, hypodermal seam cells and pharynx of the animals. The AP puncta positive for both GFP and mCherry (yellow puncta) were not easily distinguishable from the diffused fluorescence, as the mCherry fluorescence signal was much brighter than the GFP even with optimized imaging settings for both channels. As the study by Chang et al. reported that GFP positive puncta are equivalent to puncta positive for both GFP and mCherry, we similarly counted APs as GFP puncta and ALs as mCherry-only puncta (the total number of mCherry positive puncta with subtraction of the GFP positive puncta). Our control experiments by RNAi of the well-established autophagy genes *lgg-1* (homolog of atg-8) and *rab-7* (homolog of mammalian RAB7) confirmed expected results, as knockdown of *lgg-1* profoundly decreased the appearance of both APs and ALs and *rab-7* RNAi increased APs and decreased ALs in intestinal cells and hypodermal seam cells, which is consistent with its role in the fusion of autophagosomes to lysosomes (Fig. S6A). Upon *ubh-4* or *usp-14* RNAi, we observed a significant decrease in the number of APs without an effect on the number of ALs in intestinal cells (Fig. 4D). Our result suggests that in intestinal cells, the early stage of autophagy, i.e.*,* AP formation, is reduced, but not the AP fusion with the lysosome, upon downregulation of *ubh-4* or *usp-14.* Similarly, the hypodermal seam cells displayed decreased number of APs, but not ALs, when the animals were exposed to *usp-*14 RNAi, (Fig. 5A). Downregulation of *ubh-4* did not affect the number of formed APs, but resulted in an increased number of ALs, suggesting slow lysosomal degradation or faster fusion of APs with the lysosomes (Fig. 5A).

We next analyzed the effect of downregulation of *ubh-4* and *usp-14* on autophagy in the pharynx. Previous studies have reported that pharynx is resistant to first generation RNAi, but sensitive in animals exposed to RNAi for the second generation (Kumsta and Hansen, 2012; Shiu and Hunter, 2017). Accordingly, we observed no effect on the number of APs and ALs upon first generation RNAi of *lgg-1, rab-7, ubh-4* or *usp-14* (Fig. S6A,B), whereas the effect of autophagy was clearly detected in the pharynx of animals continuously exposed to *lgg-1* or *rab-7* RNAi for two generations (Fig. S6A). Animals exposed to continuous *ubh-4* or *usp-14* RNAi for two generations displayed a significant decrease in the number of APs and ALs in the pharynx (Fig. 5B).

Altogether, our results reveal that *usp-14* and *ubh-4* can have similar or distinct effects on the AP and AL stages of autophagy in the intestine, hypodermal seam cells and pharynx in *C. elegans* (summarized in Fig. 7).

### Pharmacological inhibitors of proteasome-associated DUBs affect autophagy in a tissue-specific manner in *C. elegans*

**Fig. 6.**
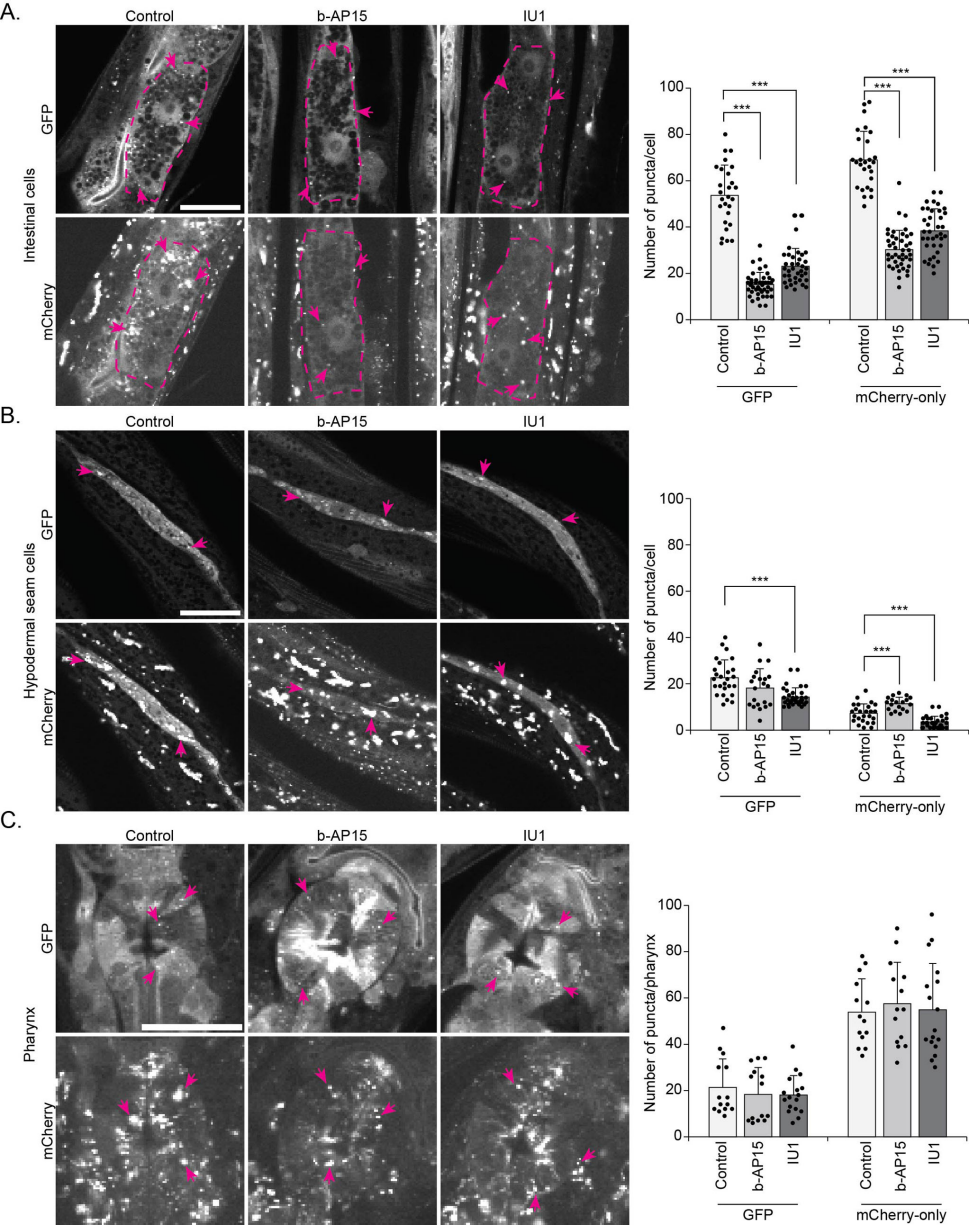
**Pharmacological inhibition of the proteasome-associated DUBs *ubh-4* and *usp-14* affects autophagy differently in different tissues.** Representative fluorescence confocal micrographs of control (DMSO), b-AP15 (10 µM) or IU1 (100 µM) treated mCherry::GFP::LGG-1 animals showing the intestinal cells (A), hypodermal seam cells (B), and the pharynx (C). Graphs show the quantification of the number of puncta positive for GFP and mCherry-only in the corresponding cells. Results are from three independent experiments. Puncta were counted from a total of 30-35 individual intestinal cells from 20-25 animals, 30-35 hypodermal seam cells from 20-25 animals and 15-17 pharynges from 15-17 animals. Error bars, STD, ***P<0.001 compared to control.

To complement the studies on genetic downregulation of *ubh-4* and *usp-14*, we also investigated the effect of the deubiquitinase inhibitors, b-AP15 and IU1, which are commonly used for mammalian cell culture studies, on autophagy in *C. elegans.* When animals expressing the mCherry::GFP::LGG-1 reporter were exposed to b-AP15 or IU1 from the L1 stage to analysis at day 1 of adulthood, we detected that both inhibitor treatments decreased the number of APs as well as ALs in the intestine (Fig. 6A). Our data reveal that pharmacological inhibitor treatment of both *ubh-4* and *usp-14* suppresses autophagy at an early stage.

**Fig. 7.**
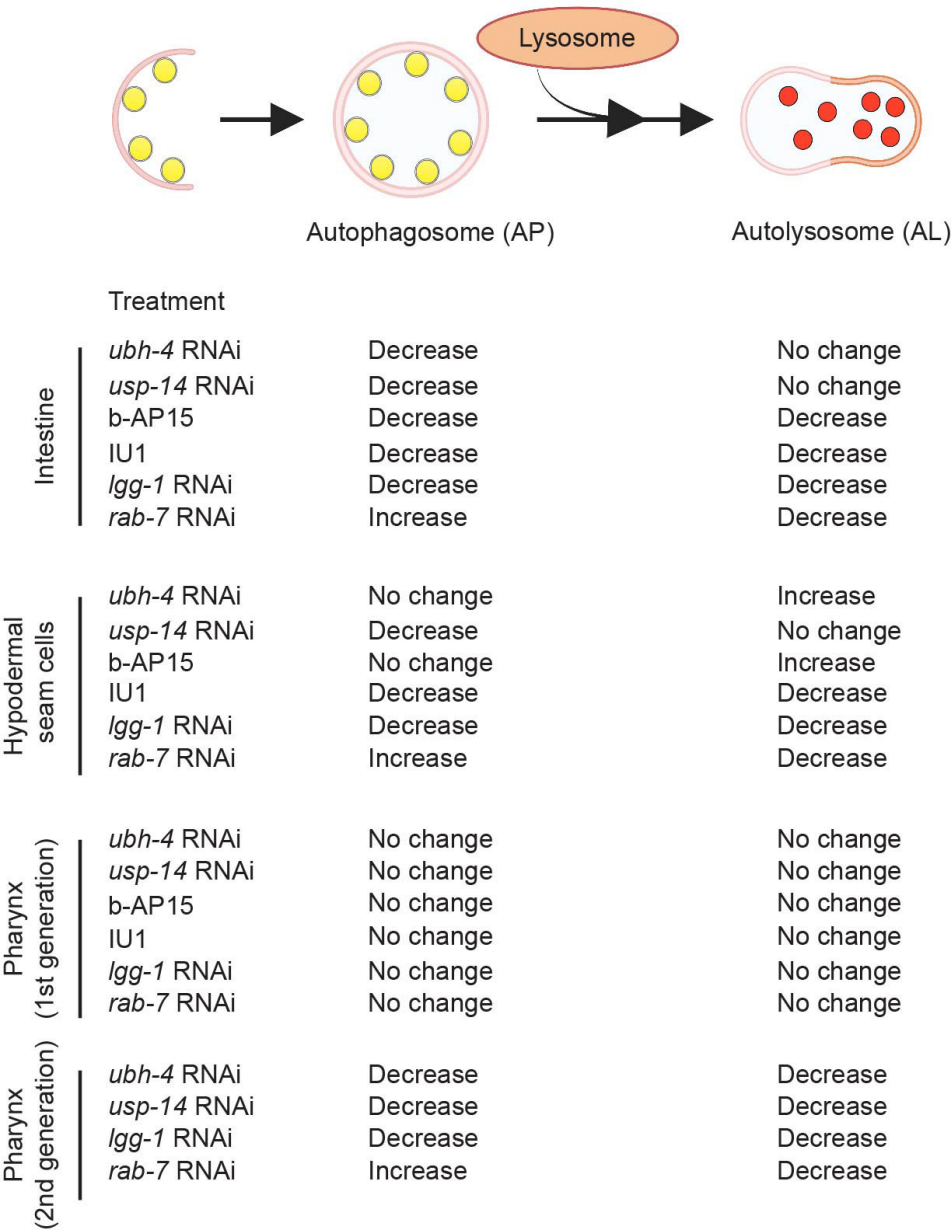
Summary of the effects of *ubh-4* and *usp-14* impairments on autophagy in the intestine, hypodermal seam cells and the pharynx.

In hypodermal seam cells, b-AP15 treatment did not affect the number of APs but resulted in more ALs (Fig. 6B) suggesting a faster fusion of APs with lysosomes or slow lysosomal degradation. The suppression of *usp-14* using IU1 decreased both APs and ALs (Fig. 6B) in hypodermal seam cells, indicating that a reduction in *usp-14* affects the early stage of autophagy. Notably, treatment with the dual inhibitor b-AP15 elicited an opposite response in terms of the effect on number of ALs. Treatments with b-AP15 and IU1 did not affect the number of APs or ALs in the pharynx (Fig. 6C).

As the inhibitors b-AP15 and IU1 affect the proteasome-associated DUBs (Lee et al., 2010), we would expect to detect a change in the accumulation of proteasomal substrates in *C. elegans*. We, therefore, used these inhibitors on our previously established fluorescent polyubiquitin reporter strain, which measures endogenous Lys-48-linked polyubiquitinated proteasomal substrates in the intestine (Matilainen et al., 2013, 2016). The polyubiquitin reporter animals were treated with b-AP15 or IU1 from the L1 larval stage until day 1 of adulthood. We detected an increase in fluorescence upon b-AP15 treatment reflecting increased accumulation of polyubiquitinated proteins in the intestine (Fig. S6A,B). In contrast, IU1 treatment resulted in decreased amount of polyubiquitinated proteins in the intestine (Fig. S7A,B). Our *C. elegans* results support previous reports on the effect of b-AP15 and IU1 on proteasomal substrates in mammalian cells (D'Arcy et al., 2011; Lee et al., 2010).

Taken together, our pharmacological data and RNAi results reveal differential effects of *ubh-4* and *usp-14* on the number of autophagosomes and autolysosomes, and a partial tissue variation, thus highlighting a complexity in the dynamics of autophagy in *C. elegans* (Fig. 7).

## DISCUSSION

In this study, we show that genetic downregulation or pharmacological impairment of the proteasome-associated DUBs Uchl5/UBH-4 and Usp14 reduces autophagy in human HeLa cells and elicits differential effects on the pool size of APs and ALs in various tissues in *C. elegans.* Our study reveals that downregulation of *Uchl5* by siRNA causes both increased GFP/RFP ratio and accumulation of LC3-II and p62 in GFP-LC3-RFP-LC3ΔG HeLa cells, suggesting a reduction in autophagy (Fig. 1). Similarly, siRNA knockdown of *Usp14* increases the GFP/RFP ratio and amount of LC3-II and p62, reflecting perturbed autophagy (Fig. S4). It is worth noticing that HeLa cells have been reported to contain high levels of LC3-II (Tanida et al., 2005), and that although LC3-II is the commonly used marker for autophagosomes, interpreting the outcome of LC3-II levels on the autophagy process is not straight forward (Klionsky et al., 2021). For example, increased LC3-II levels could be due to faster conversion from LC3-I or block in autophagosome-lysosome fusion step (Klionsky et al., 2021). As we detected accumulation of LC3-II together with p62 upon downregulation of either *Uchl5* or *Usp14*, our results show slower degradation of autophagosomal substrates indicative of reduced autophagy. The combination of our LAMP2 result, reflecting an unchanged quantity of lysosomes, with the lack of an enhancement in BAFA-induced accumulation of LC3-II and p62 in *Uchl5* or *Usp14* siRNA-treated cells compared to control siRNA-treated cells demonstrate a block in autophagosome–lysosome fusion (Fig. S3A, Fig. 2). In agreement with our Usp14 results, Kim et al. have previously reported reduced autophagic flux due to impaired fusion of autophagosome with lysosome in HEK293 cells and MEF cells upon *Usp14* downregulation (Kim et al., 2018). In contrast, downregulation of *Usp14* has also been shown to enhance autophagy in human H4 neuroglioma cells, as increased LC3-II levels concomitant with reduced p62 levels were detected (Xu et al., 2016).

As a complementary approach to the genetic downregulation of *Uchl5* and *Usp14*, we investigated the effect of the commonly used DUB inhibitors b-AP15, a dual inhibitor of Uchl5 and Usp14, and the Usp14-specific inhibitor IU1 on autophagy (Lee et al., 2010; D'Arcy et al., 2011). D'Arcy and co-workers have shown that b-AP15 functions by blocking the deubiquitinating activity of Uchl5 and Usp14 in the 19S regulatory particle of the proteasome, thereby causing accumulation of proteasomal substrates *in vitro* and in human cell lines (D'Arcy et al., 2011). IU1 treatment on the other hand has been reported to decrease the ubiquitin chain trimming capacity of Usp14 leading to increased degradation of proteasomal substrates (Lee et al., 2010). Currently, there is no commercially available inhibitor specifically targeting Uchl5 and, therefore, we used comparison studies between b-AP15 and IU1 treatments to analyze the role of Uchl5 on autophagy. Our data show that both b-AP15 treatment and IU1 treatment result in reduced autophagy, as increased number of LC3 puncta and GFP/RFP ratio as well as accumulation of LC3-II and p62 were observed in both cases (Fig. 3, Fig. S4). Thus, Uchl5 and Usp14 appear to have a similar modulatory effect on autophagy in GFP-LC3-RFP-LC3ΔG HeLa cells. It is unlikely that the impact of Usp14 on autophagy would override that of Uchl5, as our siRNA results show that both Usp14 and Uchl5 knockdown reduces autophagy. Supportive results on LC3-II accumulation upon b-AP15 treatment have previously been shown in triple negative breast cancer (TNBC) cell lines (Vogel et al., 2015). It has also been reported that the DUB inhibitor NiPT, which also blocks both Uchl5 and Usp14, induces autophagy in A549 and NCI-H1299 lung cancer cell lines (Chen et al., 2020). In agreement with a previous study (Kim et al., 2018), we demonstrate that IU1 treatment impairs autophagy, as detected by increased number of LC3 puncta and GFP/RFP ratio as well as accumulation of LC3-II and p62. A more complex view on the effect of IU1 treatment on autophagy is described by Xu and colleagues (Xu et al., 2020) by showing that lower concentration increases the levels of both LC3-II and p62, whereas higher concentration increases LC3-II but decreases p62 levels in HeLa cells. In our case, we detected increased levels of both LC3-II and p62 upon treatment with a similar high concentration of IU1, which could be due to different treatment duration and/or the transgenic GFP-LC3-RFP-LC3ΔG HeLa cell line. A study by Srinivasan and colleagues reveals that IU1 treatment differentially influences autophagy flux i.e., the levels of LC3-II upon blockage of fusion of autophagosomes with lysosomes, in ML1 and primary thyroid cells, but without a concurrent effect on p62 levels (Srinivasan et al., 2023).

Our results extend further support for a functional interplay between UPS and ALP, where impairment or activation of proteasome has previously been reported to cause induction or reduction of autophagy, respectively. For instance, pharmacological inhibition of the proteasome by lactacystin enhances autophagy in SH-SY5Y neuroblastoma cells and in a UPS-compromised mice model (Shen et al., 2013). Similarly, treatment with the MG-132 proteasome inhibitor activates autophagy in rat alveolar macrophage cells (Fan et al., 2016) and in HEK293 cells (Li et al., 2019). Pharmacologically or genetically induced impairment of the proteasome enhances autophagy in the cardiomyocytes of mice (Zheng et al., 2011; Pan et al., 2020), and RNAi targeting of different proteasome subunits results in both enhanced basal autophagy as well as starvation-induced autophagy in *Drosophila* larvae (Lőw et al., 2013). Conversely, stimulation of proteasomal substrate degradation through downregulation of Usp14 results in reduced autophagic flux in HEK293 and MEF cells (Kim et al., 2018). Further, increased proteasome levels and activity correlate with reduced autophagy in the retina of rhodopsin P23H mutant mice treated with the phosphodiesterase-4 inhibitor rolipram (Qiu et al., 2019). In this study, we show that modulation of the proteasome via genetic downregulation or pharmacological inhibition of the proteasome-associated DUBs *Uchl5* or *Usp14* causes reduced autophagy in human GFP-LC3-RFP-LC3ΔG HeLa cells (Figs 1,3, Fig. S4).

Our results also reveal how modulating UPS via the proteasome-associated DUBs Uchl5/UBH-4 and USP-14 affect autophagy at the tissue level in *C. elegans.* Autophagy plays a key role in various developmental and physiological processes in *C. elegans* including embryogenesis, development, dauer formation, longevity, and stress responses (Meléndez et al., 2003; Hansen et al., 2008; Zhao et al., 2009; Tian et al., 2010; Alberti et al., 2010; Wu et al., 2015; Palmisano and Meléndez, 2019; Chen et al., 2021). In adult animals, autophagy has been previously investigated in intestinal cells, hypodermal seam cells, neurons, muscle cells, and the pharynx (Chapin et al., 2015; Zhang et al., 2015; Chang et al., 2017; Zheng et al., 2020). We show that downregulation of *ubh-4* or *usp-14* by RNAi has different effects on the pool size of AP and AL in intestinal cells, hypodermal-seam cells, and pharynx (Figs 4, 5 and 7). The *usp-14* RNAi appears to reduce autophagosome formation both in the intestinal and hypodermal seam cells without affecting the rate of autophagosome–lysosomal fusion, and *ubh-4* knockdown has a similar effect in intestinal cells. However, in hypodermal seam cells *ubh-4* downregulation leads to increased number of autolysosomes without a change in autophagosome number, suggesting that *ubh-4* downregulation slows the rate of lysosomal degradation or potentially induces a faster fusion of autophagosomes to lysosomes in this tissue. The *ubh-4* RNAi results from the analyzed tissues in *C. elegans* are not in line with the *Uchl5* siRNA results, suggesting a tissue context dependency in autophagy responses upon *ubh-4* downregulation in a multicellular organism. In support of tissue differential autophagy responses *in vivo*, the autophagy block upon *Usp14* downregulation in the HeLa cells (Fig. 2) as well as in HEK293 and USP14^−/−^ MEF cells (Kim et al., 2018) differs from our analyzed tissues in *C. elegans* (Figs 4D, 5A). In agreement with a tissue differential effect of DUBs on autophagy, several studies have reported that autophagy varies in different tissues in *C. elegans.* According to Chapin et al. there are cell type-specific differences in the overall rate of autophagic flux both in basal and stress-induced autophagy in *C. elegans* (Chapin et al., 2015), and tissue variations in the age-dependent decrease in autophagy have been reported (Chang et al., 2017). Zheng et al. have revealed that the autophagy genes are involved in a tissue- and stage-specific manner during the development of *C. elegans* (Zheng et al., 2020). Additionally, dietary restriction has been shown to result in reduction of the number of autophagosomes in intestinal cells (Gelino et al., 2016).

Compared to the RNAi-induced autophagy response in the intestine and hypodermal seam cells, the same animals did not display any phenotype in the pharynx after the 3 days of RNAi treatment, not even when targeting *lgg-1* or *rab-7* (Fig. S6A,B). However, continuous exposure of the animals to *ubh-4* or *usp-14* RNAi treatments for two generations resulted in a decreased number of autophagosomes and autolysosomes (Fig. 5B), suggesting a reduced autophagic flux. Thus, similar to the intestinal and hypodermal seam cells results, downregulation of *ubh-4* in pharynx does not appear to block the lysosomal fusion step, in contrast to the siRNA results in HeLa cells. Previous studies have also reported that pharynx is resistant to first generation RNAi, but sensitive in animals upon continuous RNAi feeding for two generations (Kumsta and Hansen, 2012; Shiu and Hunter, 2017). Downregulation by RNAi has its limitations, but in comparison to a potential compensatory redundancy and a chronic effect in knockout animals, RNAi and/or pharmacological inhibition studies offer a way to mimic a stress or disease condition.

In agreement with our RNAi studies targeting *ubh-4* and *usp-14,* pharmacological inhibition of these DUBs induced variation in the pool size of autophagosomes and autolysosomes in different tissues in *C. elegans* (Fig. 6). For a validation study, we tested these inhibitors on a previously established polyubiquitin reporter strain and observed that b-AP15 treatment leads to increased fluorescence indicating enhanced accumulation of polyubiquitinated proteins (Fig. S7A,B), whereas animals treated with IU1 caused decreased amount of polyubiquitinated proteins in the intestine. These results are in agreement with the human cell line data, where accumulation of proteasomal substrates upon b-AP15 treatment and increased degradation of proteasomal substrates upon IU1 treatment have been shown (Lee et al., 2010; D'Arcy et al., 2011). Our b-AP15 results on polyubiquitin accumulation differ from our previously published result on the effect of *ubh-4* RNAi which showed that downregulation of *ubh-4* results in decreased accumulation of the polyubiquitin reporter, and increased proteasome activity *in vivo* and *in vitro* (Matilainen et al., 2013). The difference between the b-AP15 and *ubh-4* RNAi effect is likely due to the dual inhibitory effect of b-AP15 on UBH-4 and USP-14.

Monitoring autophagy in an adult multicellular organism is intricate due to the diverse and morphologically distinct cell types with spatial and temporal differences in responses to physiological or pathophysiological stress conditions. Altogether, our data reveal that modulation of UPS via pharmacological or genetic impairment of the proteasome-associated DUBs UBH-4 and USP-14 differentially affects autophagy in a tissue-variable manner but appears not to block autophagosome–lysosomal fusion. However, future studies are required to reveal the molecular mechanisms underlying the tissue-specific differences in autophagy in response to Uchl5/UBH-4 and Usp14. Our results thus expand the existing knowledge on regulation of autophagy in different cell types (Klionsky et al., 2021) and emphasize the dynamic complexity of autophagy *in vivo.* Importantly, our study highlights the context dependency of autophagy regulation in a multicellular organism in comparison to *in vitro* studies in cell lines. More detailed information on the specificity in tissue regulation of autophagy will promote development of therapeutic options targeting autophagy or autophagy-associated diseases.

## MATERIAL AND METHODS

### Mammalian cell cultures

HeLa cells expressing GFP-LC3-RFP-LC3ΔG were obtained from Riken BRC cell bank (resource number RCB4695) and the autophagy reporter function verification is included in this manuscript. The cells were cultured in high glucose DMEM. The media was supplemented with 10% fetal bovine serum (FBS), L-glutamine, penicillin and streptomycin and maintained in a 5% CO2 incubator. For siRNA experiments, FlexiTube GeneSolution for *Uchl5* and *Usp14* (QIAGEN) and AllStars Negative Control siRNA (QIAGEN) were used with HiPerFect transfection Reagent (QIAGEN). Cells treated with siRNA were incubated for 48 h prior to live imaging or sample collection for quantitative analysis. For pharmacological treatment, we used 1 uM of b-AP15 for 18 h (Sigma-Aldrich, 662140), 100 μM IU1 for 6 h (Sigma-Aldrich, 662210) or 100 nM BAFA for 6 h (Sigma-Aldrich, B1793). DMSO (Thermo Fisher Scientific, 15498089) was used as vehicle control.

### Immunofluorescence

GFP-LC3-RFP-LC3ΔG expressing HeLa cells transfected with control, *Uchl5* or *Usp14* siRNA were grown on cover slips for 48 h. Cells were fixed with 4% parafolmaldehyde (PFA) (Electron Microscopy Sciences, 19208) in phosphate buffered saline (PBS) (Lonza, BE17-516F) followed by quenching of residual PFA with 50 mM NH_4_Cl. The fixed cells were permeabilized with 0.1% Triton X-100 in PBS before mounting with SlowFade Diamond Antifade Mountant (Thermo Fisher Scientific, Waltham, MA, USA, S36967). Primary antibody anti-goat LAMP2 (R&D, AF6228) was used in 1:100 dilution. The donkey anti-goat alexa fluor 647 conjugated secondary antibody (Invitrogen, A21447) in 1:200 dilution was used for visualization.

### Microscopy, equipment, and image analysis

GFP-LC3-RFP-LC3ΔG expressing HeLa cells transfected with control, *uchl5* or *usp14* siRNA or treated with inhibitors were grown in Thermo Scientific™ Nunc™ Lab-Tek™ II chambered coverglass plates (Thermo Fisher Scientific, 16260671) and imaged after 48 h. For autophagy flux measurement cells were transfected with control, *Uchl5* or *Usp14* siRNA for 48 h, and treated with BAFA for the last 6 h prior to imaging. Live cells were imaged with a Zeiss LSM880 confocal microscope (Motorized Zeiss Axio Observer.Z1 inverted microscope), at 63×1.4 NA plan-Apochromat objective and at 37°C and 5% CO_2_. Confocal images were converted to tiff-format using Zen 2 lite (blue). Images were quantified from the original version without any modification, using Fiji ImageJ software. The number of puncta was counted using plugins, FeatureJ, and FeatureJ Laplacian commands from Fiji software. A threshold was selected so that most of the dots were selected for all images, and then the analyze particle command gave the number of puncta present on that image. For quantification of the total fluorescence intensity of images, we used Fiji ImageJ software. The background was subtracted using the corresponding command in Fiji software. Threshold was selected for the brightest images and the same threshold was applied to all images from the same experiment. The average of mean intensity was analyzed.

All the images were processed with Fiji ImageJ software. The brightness of the images was increased in the same way to all corresponding images from the same experiment, to be able to make the fluorescent signal clearly visible.

### Western blotting

For Western blotting, cell lysates were collected after 48 h of siRNA treatment, 18 h of b-AP15 or 6 h of IU1 treatment. For autophagy flux measurement cells were transfected with control, *Uchl5* or *Usp14* siRNA for 48 h and treated with BAFA for the last 6 h prior to collecting cells for Western blot analysis. The cells were lysed by vigorous vortexing. Samples were run on SDS-PAGE gel and immunoblotted onto a nitrocellulose membrane using Trans-Blot Turbo transfer system (Bio-Rad). Anti-LC3B antibody (anti-rabbit, Sigma-Aldrich, L7543, 1:5000 dilution), anti-P62 antibody (anti-rabbit, Sigma, P0067, 1:10,000 dilution), anti-UCHL5 (anti-mouse, Santa Cruz, sc271002, 1:500 dilution), anti-USP14 (anti-mouse, Sigma, SAB1406778, 1:2500 dilution) and anti-HSC70 antibody (anti-mouse, Santa Cruz, sc7298, 1:5000 dilution) were used for immunoblotting. For the anti-LC3B antibody and the anti-P62 antibody, the secondary antibody used was anti-rabbit IgG-HRP conjugate (LA_W4011, 1:10,000 dilution), and for anti-UCHL5, anti-USP14 and HSC70 anti-mouse IgM-HRP conjugate (Calbiochem, 401225, 1:10,000 dilution). Image Studio software (Licor) was used for imaging and quantifying the signals.

### Quantitative real-time PCR

Cell lysates were collected after 48 h of siRNA treatment and stored at −80°C. Total RNA was extracted from the freezed samples using NucleoSpin RNA kit (Macherey-Nagel) and concentration of extracted RNA was measured with Nanodrop spectrophotometer at 260 nm. RT-PCR was performed using Maxima First Standard cDNA Synthesis Kit for RT-qPCR (Fermentas). The quantitative real-time PCR was done using Maxima SYBR Green/ROX qPCR Master Mix (2×) (Fermentas) and LightCycler 480 (Roche) quantitative PCR machine. The data from qPCR were normalized to the geometric mean of mRNA concentration of two reference genes (*gapdh* and *cyclophilin*).

### *C. elegans* and growth conditions

*C. elegans* strains were cultured and maintained as described previously (Brenner, 1974) under standard conditions at 20°C, on nematode growth medium (NGM) plates seeded with OP50. N2 (Bristol) and MAH215 were obtained from the Caenorhabditis Genetics Center (CGC). Only hermaphrodites were used for the analysis.

### *C. elegans* RNA interference (RNAi)

RNAi was performed using the feeding method as described earlier (Timmons et al., 2001). The HT115 bacterial strain carrying the empty *pL4440* expression vector was used as a control. RNAi clones used in this study were from J. Ahringer library. The double stranded RNA expression was induced by adding 0.4 mM of isopropyl-β-D-thiogalactopyranoside (IPTG) (I6758, Sigma) and its concentration was further increased to 0.8 mM prior to seeding the plates. Unless otherwise indicated, age-synchronized animals were placed on control as well as RNAi seeded plates targeting *ubh-4* and *usp-14* as L1 larvae (day 1). For imaging experiments, *ubh-4* and *usp-14* RNAi-treated animals were imaged at first day of adulthood (day 4).

### Inhibitor treatment

Chemicals were added to the nematode growth medium (NGM) prior pouring to the plates. We used DMSO (Thermo Fisher Scientific, 15498089) as a control, 10 μM of b-AP15 (Sigma-Aldrich, 662140) or 100 μM IU1 (Sigma-Aldrich, 662210). NGM plates with chemicals were seeded with OP50 Escherichia coli bacteria. Once the plates were dried, age synchronized animals were placed on chemical seeded plates on the same day as L1 larvae (day 1). Chemical treated animals were imaged at first day of adulthood (day 4).

### Microscopy of *C. elegans* and quantitative image analysis

Age synchronized animals were imaged at the first day of adulthood (day 4). Animals were mounted on 3% agarose pad on glass slides and immobilized using 1 mM levamisole in M9 buffer (22 mM KH_2_PO_4_, 41 mM Na_2_HPO_4_, 8,5 mM NaCl and 19 mM NH_4_Cl). Autophagy dual marker reporter strains were imaged using LSM780 confocal microscope (Motorized Zeiss Axio Observer.Z1 inverted microscope), z-stack images were acquired at 0.8 µm slice intervals at 40×1.3 NA plan Neofluor objective. Z-stack images were converted to maximum intensity projection format using ZEN 2.1 (black) and converted to tiff-format using Zen 2 lite (blue). The number of puncta was calculated manually.

All the images were processed with Fiji ImageJ software. The brightness of the images was increased in the same way to all corresponding images from the same experiment, to be able to make the fluorescent signal clearly visible.

### Quantitative real-time PCR

Age synchronized RNAi-treated animals were collected in M9 at first day of adulthood (day 4) and stored at −80°C. Total RNA was extracted from the frozen samples using NucleoSpin RNA kit (Macherey-Nagel) and concentration of extracted RNA was measured with Nanodrop spectrophotometer at 260 nm. RT-PCR was performed using Maxima First Standard cDNA Synthesis Kit for RT-qPCR (Fermentas). The quantitative real-time PCR was done using Maxima SYBR Green/ROX qPCR Master Mix (2×) (Fermentas) and LightCycler 480 (Roche) quantitative PCR machine. The data from qPCR were normalized to the geometric mean of mRNA concentration of three reference genes (*act-1, cdc-42* and *pmp-3*) (Vandesompele et al., 2002).

### Statistical analysis

Statistical significance for all experiments was determined using both the Student's *t*-test (two-tailed) and the one-way ANOVA. All analyses were done on raw data and both tests gave the similar statistical significance. The number of animals analyzed in each experiment are described in the figure legends.

## Supplementary Material

10.1242/biolopen.061644_sup1 Supplementary information
