# Supplementary material for: Impairment of proteasome-associated deubiquitinating enzyme Uchl5/UBH-4 affects autophagy
Source: Biol Open. 2025 Feb 6;14(2):bio061644. doi: 10.1242/bio.061644 (PMC11832120; doi:10.1242/bio.061644)
Supplement: Supplementary information [file biolopen-14-061644-s1.pdf]

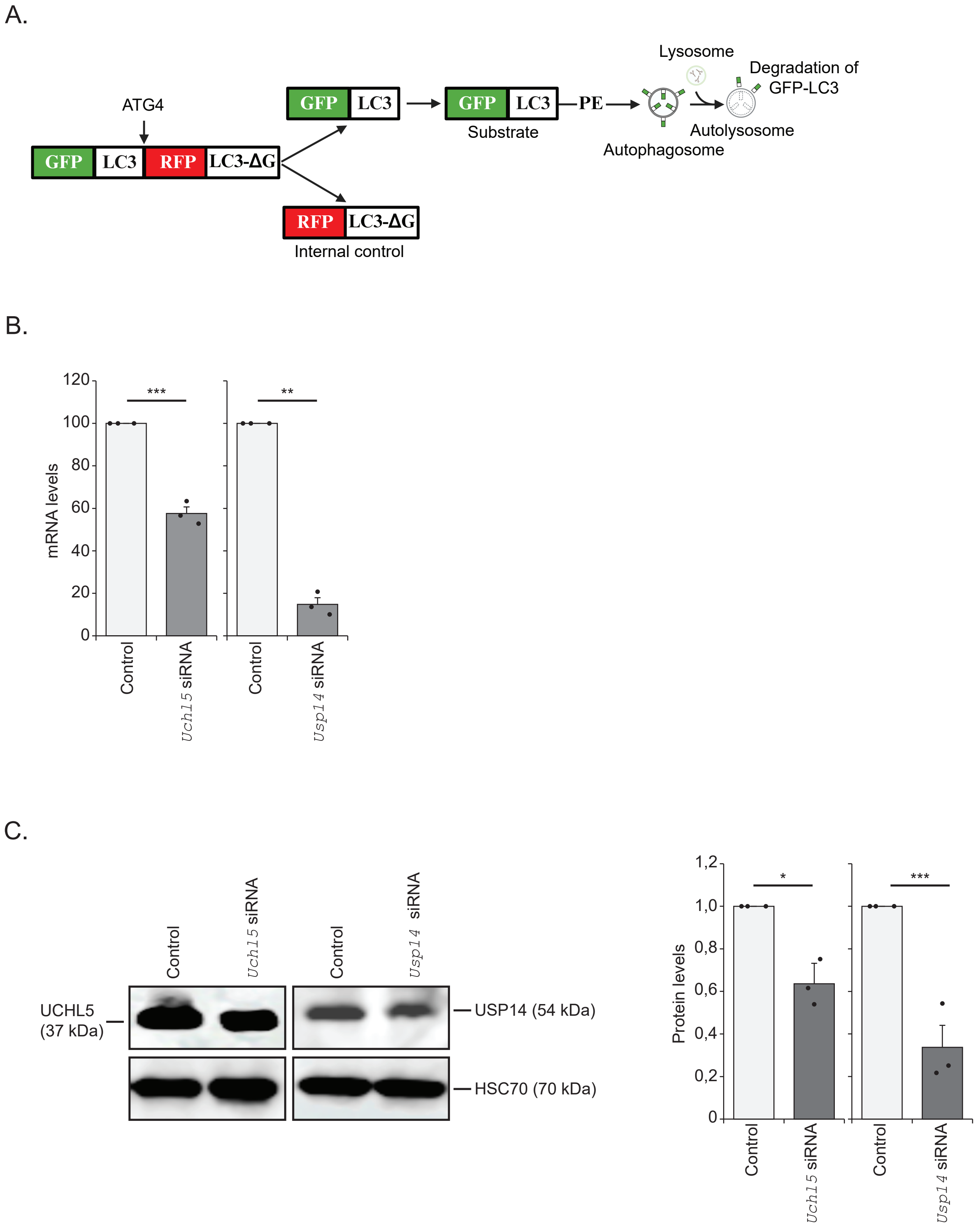

Fig. S1. Downregulation of *Uchl5* and *Usp14* upon treatment with siRNA. A. Schematic representation of the GFP-LC3-RFP-LC3ΔG fluorescent probe (Kaizuka et al., 2016). **B.** GFP-LC3-RFP-LC3ΔG HeLa cells treated with control, *Uchl5* or *USsp14* siRNA and collected 48h post-transfection. Expression of *Uchl5* or *Usp14* mRNA was measured with qPCR. Graph shows the percentage change in the mRNA levels compared to control (set as 100%). Results are the mean of quantifications from three independent experiments. Error bars, SEM, **p<0,01, ***p<0,001 compared to the control (set as 100). **C.** Whole cell extracts (48h post-transfection) were analyzed by SDS-PAGE and immunoblotted against Uchl5, Usp14 and HSC70. The graphs (on right panel) show average fold change in levels of Uchl5 and Usp14 normalized against HSC70. Results are the mean of quantifications from three independent experiment. Error bars, SEM, *p<0,05, ***p<0,001 compared to the control (set as 1).

Biology Open • Supplementary information

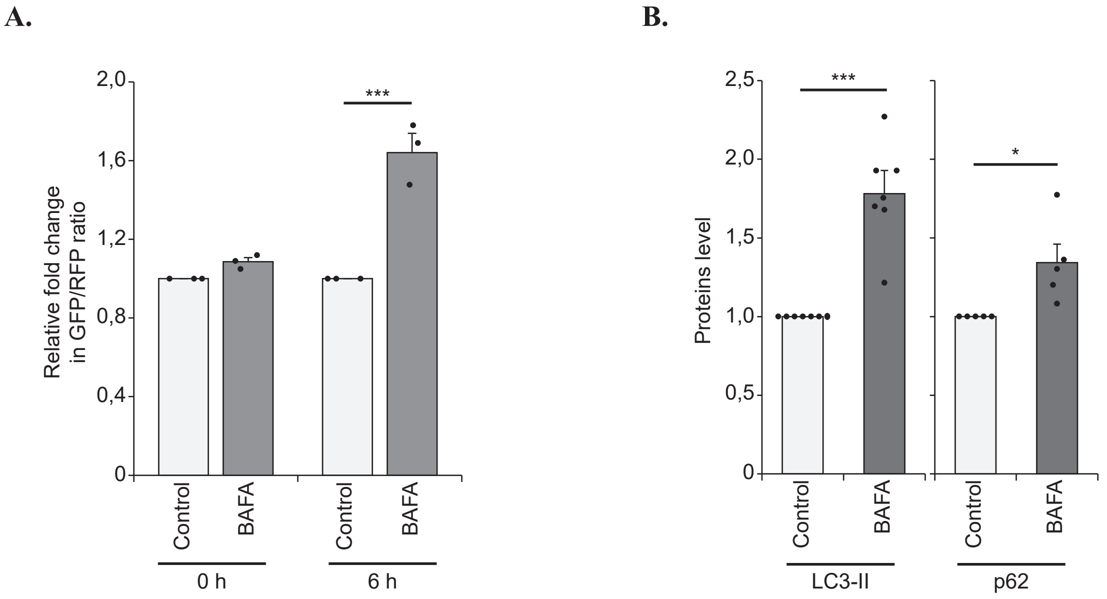

Fig. S2. Validation experiments of the GFP-LC3-RFP-LC3ΔG HeLa cell line using BAFA. A. GFP-LC3-RFP-LC3ΔG HeLa cells treated with control (DMSO) or BAFA (100 nM) for 6h. The graph shows the quantification of the relative fold change in the ratio of GFP/RFP per image (Control set at 1) Results are from three independent experiments (a total of 15 images per treatment were analyzed). Error bars, SEM, ***p<0,001 compared to control. **B.** GFP-LC3-RFP-LC3ΔG HeLa cells treated with control (DMSO) or BAFA (100 nM) for 6h. Whole cell lysates were analyzed by SDS-PAGE and immunoblotted against LC3-II, p62 and HSC70. The graphs show average fold change in levels of LC3-II and p62 normalized against HSC70. Results are the mean of quantifications from three independent experiments. Error bars, SEM, *p<0,05, ***p<0,001 compared to the control (set as 1).

Biology Open • Supplementary information

A.

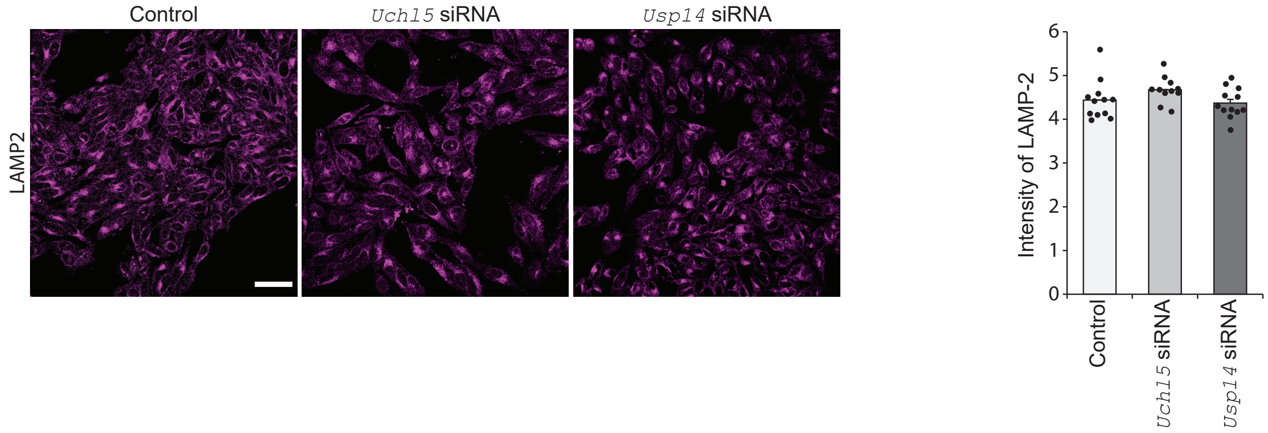

Fig. S3. Downregulation of *Uchl5* or *Usp14* does not affect the amount of LAMP2 (lysosome-associated membrane protein 2). Representative immunofluorescence confocal images of LAMP2 (magenta color) in control, *Uchl5* or *Usp14* siRNA treated GFP-LC3-RFP-LC3ΔG HeLa cells 48h post-transfection. Scale bar, 20 µm. The graph (right) shows quantification of the change in fluorescence intensity of LAMP2 per image. Results are from two independent experiments (a total of 11-12 images per treatment were analyzed). Error bars, SEM, p-value was not significant.

Biology Open • Supplementary information

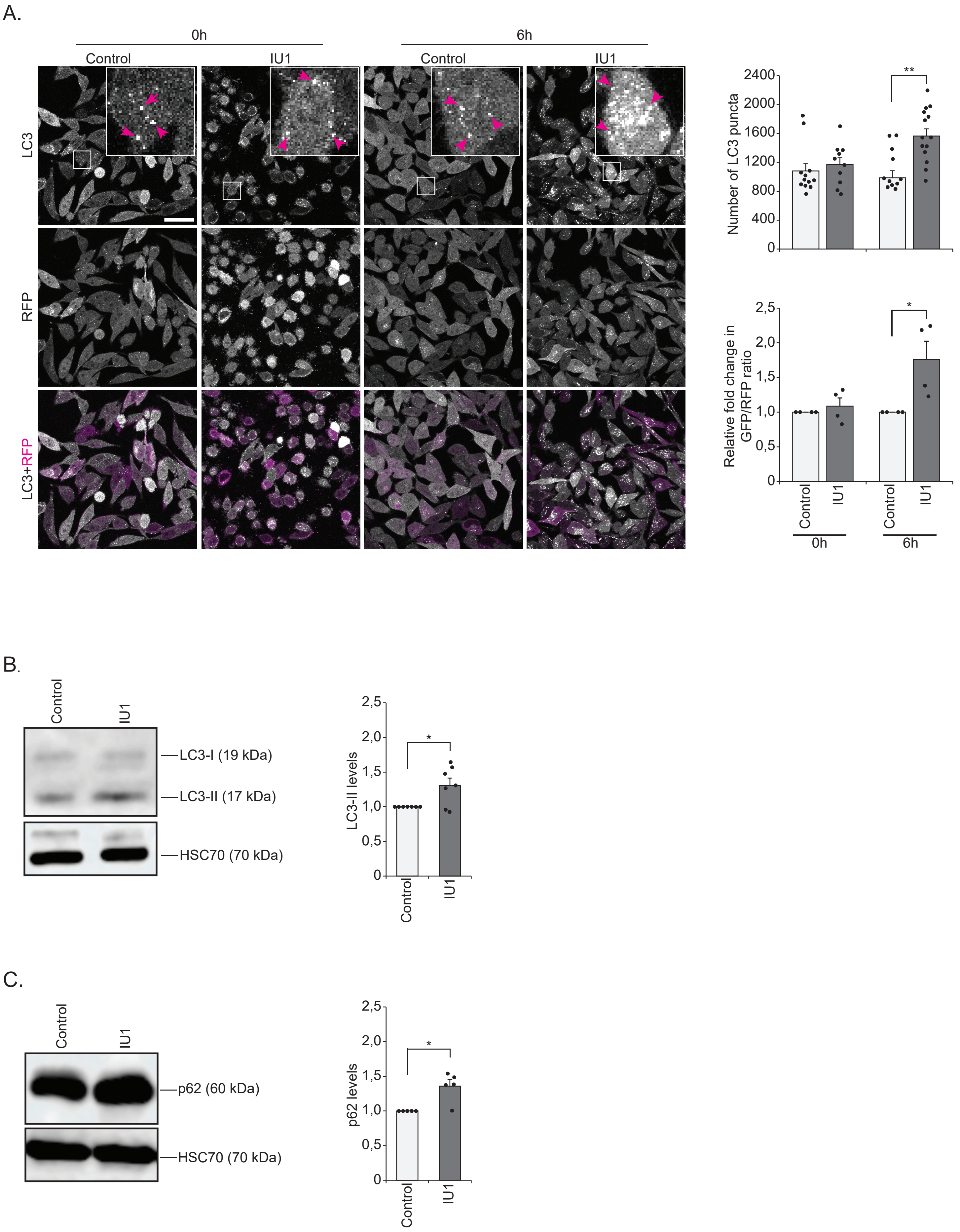

Fig. S4. **Pharmacological inhibition of Usp14 using IU1 inhibitor reduces autophagy. A.** Fluorescence confocal images of control (DMSO) or IU1 (100µM) treated GFP-LC3-RFP-LC3ΔG HeLa cells after 6h post-treatment. Insets show enlarged view of the indicated areas. Magenta arrows point to some of the puncta. Scale bar, 20 µm. The right upper graph shows the quantification of the number of GFP-LC3 puncta per image. The right lower graph shows the quantification of the relative fold change in the ratio of GFP to RFP per image (Control set at 1). Results are from four independent experiments (total 15-20 images were analyzed). Error bars, SEM, *p<0,05, **p<0,01 compared to control. **B and C**. GFP-LC3-RFP-LC3ΔG HeLa cells treated with control or IU1 (100µM) for 6h. Whole cell lysates were analyzed by SDS-PAGE and immunoblotted against LC3-II, p62 and HSC70. The graphs (on right panel) show average fold change in levels of LC3-II (B) and p62 (C) normalized against HSC70. Results are the mean of quantifications from six independent experiments. Error bars, SEM, *p<0,05 compared to the control (set as 1).

Biology Open • Supplementary information

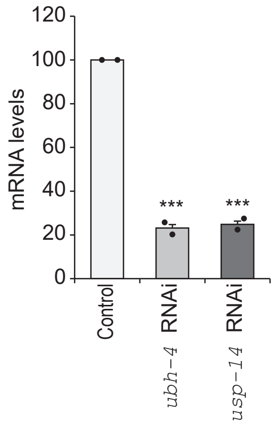

Fig. S5. Efficient downregulation of *ubh-4* and *usp-14* upon RNAi. Wild-type animals were exposed to control, *ubh-4* or *usp-14* RNAi treatment starting at the L1 larval stage and collected at Day 1 of adulthood. Expression of *ubh-4* or *usp-14* mRNA in the RNAi-treated animals was checked with qPCR. Graph shows percentage change in mRNA levels compared to control (set as 100%). The results are the mean of two independent experiments in triplicate. Error bar, SEM, ***p<0,001 compared to control.

Biology Open • Supplementary information

A.

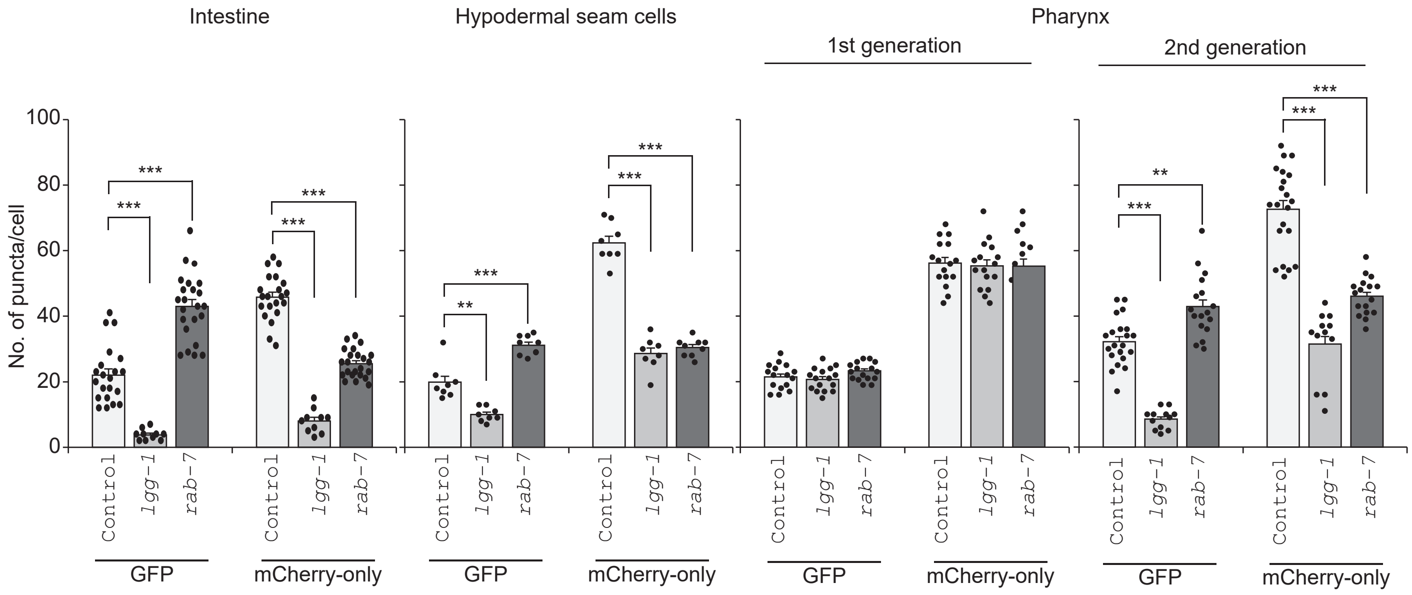

B.

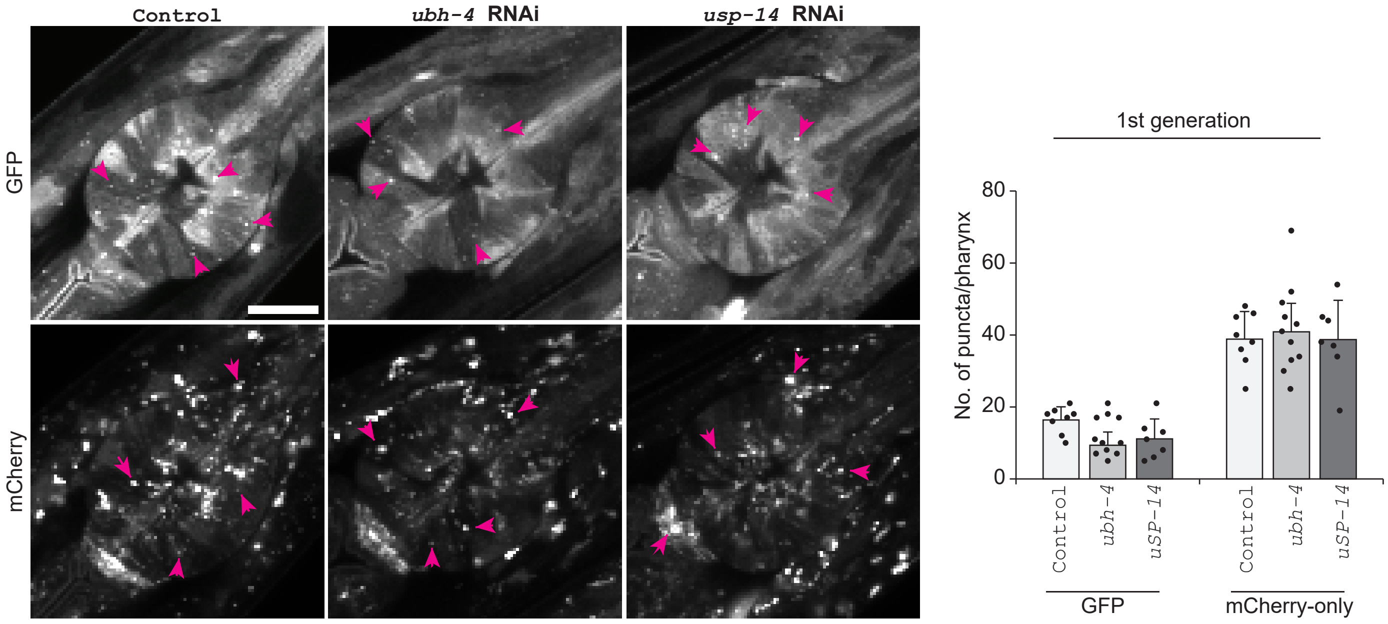

Fig. S6. Effect of downregulation of autophagy genes or proteasome-associated DUBs on autophagy in different tissues. A Graphs show the quantification of the number of puncta positive for GFP and mCherry-only in the intestinal cells, hypodermal seam cells and the pharynx. All animals, except the ones labelled second generation, were exposed to RNAi from L1 larval stage to day 1 of adulthood (First generation). The animals and their progeny were further continuously exposed to RNAi, and the day 1 of adulthood of the progeny is here labelled as second generation. Results are from three independent experiments. Puncta were counted from a total of 20-30 individual corresponding cells. Error bars, SEM, **p<0,01, ***p<0,001 compared to control. **B.** Fluorescence confocal micrographs of control, *ubh-4* or *usp14* RNAi-treated mCherry::GFP::LGG-1 animals showing the pharynx at day 1 of adulthood (First generation)**.** Graphs show the quantification of the number of puncta positive for GFP and mCherry-only. Results are from three independent experiments. Puncta were counted from a total of 10-12 pharynges from 10-12 animals. Error bars, SEM.

Biology Open • Supplementary information

A.

B.

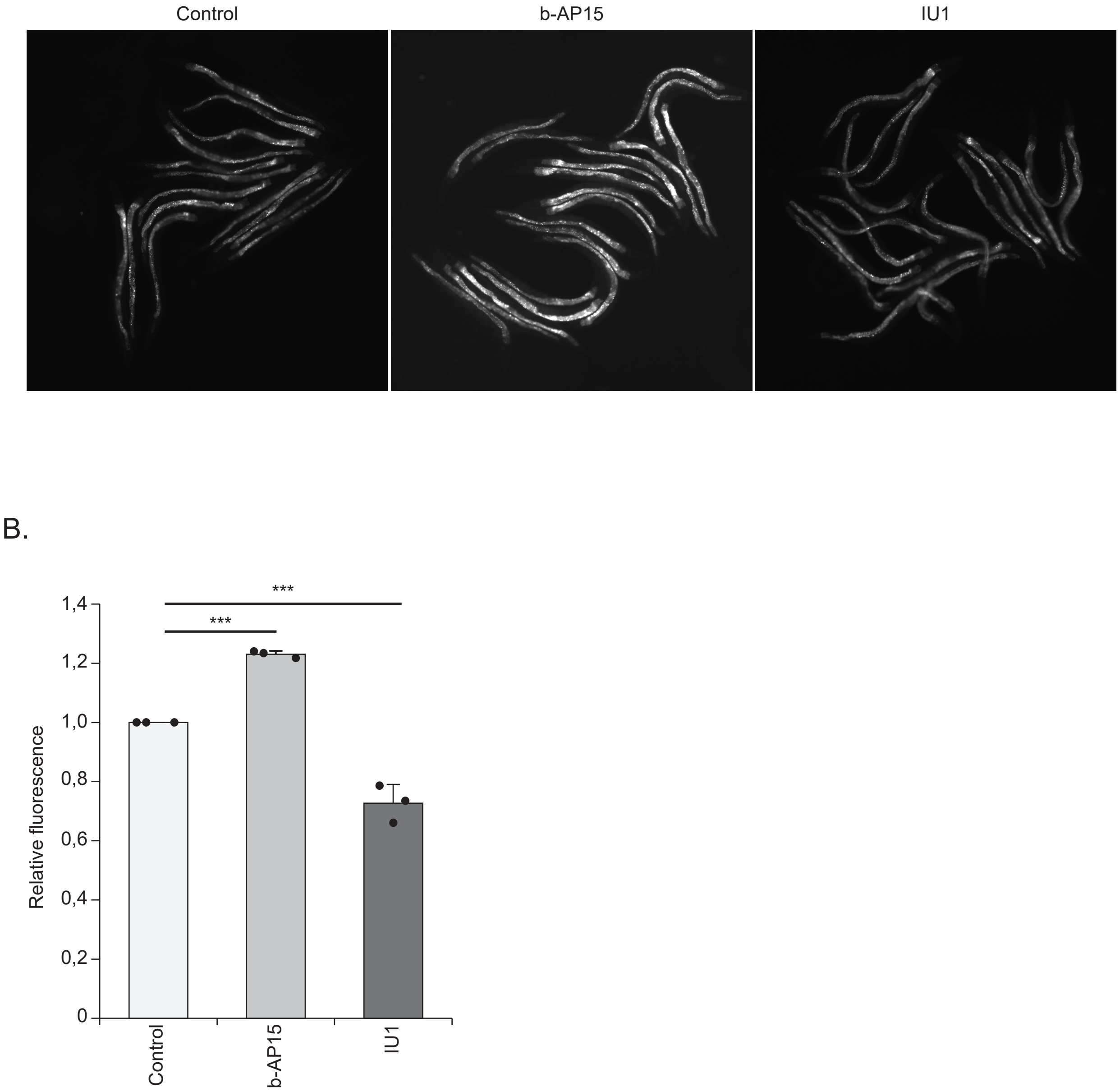

Fig. S7. The b-AP15 and IU1, inhibitors of the proteasome-associated DUBs UBH-4 and USP-14, affect accumulation of polyubiquitinated proteins in intestinal cells. A. Representative fluorescence micrographs of control (DMSO), b-AP15 (10 μM) or IU1 (100 μM) treated animals expressing the polyubiquitin reporter in the intestinal cells. Animals were treated with the inhibitors from L1 larval stage till day 1 of adulthood. **B.** Graph shows quantification of fluorescence, which reflects accumulation of polyubiquitinated proteins in the intestinal cells. Results are from three independent experiments (number of animals 90- 100). Error bars, SEM, ***p<0,001 compared to control.

Biology Open • Supplementary information
